# Protective Actions of Anserine Under Diabetic Conditions

**DOI:** 10.3390/ijms19092751

**Published:** 2018-09-13

**Authors:** Verena Peters, Vittorio Calabrese, Elisabete Forsberg, Nadine Volk, Thomas Fleming, Hans Baelde, Tim Weigand, Christian Thiel, Angela Trovato, Maria Scuto, Sergio Modafferi, Claus Peter Schmitt

**Affiliations:** 1 Centre for Paediatric and Adolescent Medicine, University Hospital of Heidelberg, 69120 Heidelberg, Germany; tim.weigand@med.uni-heidelberg.de (T.W.); christian.thiel@med.uni-heidelberg.de (C.T.); sergio.modafferi@gmail.com (S.M.); clauspeter.schmitt@med.uni-heidelberg.de (C.P.S.); 2 Department of Biomedical and Biotechnological Sciences, School of Medicine, University of Catania, 95123 Catania, Italy; calabres@unict.it (V.C.); trovato@unict.it (A.T.); scuto@unict.it (M.S.); 3 Department of Molecular Biosciences, The Wenner-Gren Institute, Stockholm University, SE-106 91 Stockholm, Sweden; elisabete.forsberg@su.se; 4 Department of Medicine I and Clinical Chemistry, University Hospital of Heidelberg, 69120 Heidelberg, Germany; Nadine.Volk@med.uni-heidelberg.de (N.V.); thomas.fleming@med.uni-heidelberg.de (T.F.); 5 Department of Pathology, Leiden University Medical Center, 2300RC L1Q Leiden, The Netherlands; J.J.Baelde@lumc.nl

**Keywords:** diabetes, diabetic nephropathy, anserine, carnosine, Hsp70, proteinuria, vascular permeability

## Abstract

Background/Aims: In rodents, carnosine treatment improves diabetic nephropathy, whereas little is known about the role and function of anserine, the methylated form of carnosine. Methods: Antioxidant activity was measured by oxygen radical absorbance capacity and oxygen stress response in human renal tubular cells (HK-2) by RT-PCR and Western-Immunoblotting. In wildtype (WT) and diabetic mice (db/db), the effect of short-term anserine treatment on blood glucose, proteinuria and vascular permeability was measured. Results: Anserine has a higher antioxidant capacity compared to carnosine (*p* < 0.001). In tubular cells (HK-2) stressed with 25 mM glucose or 20–100 µM hydrogen peroxide, anserine but not carnosine, increased intracellular heat shock protein (Hsp70) mRNA and protein levels. In HK-2 cells stressed with glucose, co-incubation with anserine also increased hemeoxygenase (HO-1) protein and reduced total protein carbonylation, but had no effect on cellular sirtuin-1 and thioredoxin protein concentrations. Three intravenous anserine injections every 48 h in 12-week-old db/db mice, improved blood glucose by one fifth, vascular permeability by one third, and halved proteinuria (all *p* < 0.05). Conclusion: Anserine is a potent antioxidant and activates the intracellular Hsp70/HO-1 defense system under oxidative and glycative stress. Short-term anserine treatment in diabetic mice improves glucose homeostasis and nephropathy.

## 1. Introduction

L-Carnosine (ß-alanyl-L-histidine) and L-anserine (ß-alanyl-Np-methyl-L-histidine) belong to the group of histidine-containing dipeptides (HDPs). HDPs are present in humans, mammals, fish and amphibia and their ratio and concentrations vary greatly among different species and organs. In mammals and humans, carnosine is the most prominent dipeptide, with highest concentrations in the muscle, together with either anserine or ophidine at different ratios [1]. Carnosine is synthesized by carnosine synthase (EC 6.3.2.11) and anserine by methylation of carnosine via carnosine N-methyltransferase (EC 2.1.1.22) [2]. In addition to muscle tissue, carnosine is mainly present in the brain and kidney of mice and humans [1]. Carnosine and anserine can be degraded by carnosinase 1 (CN1, EC 3.4.13.20) and at high pH by carnosinase 2 (CN2, EC 3.4.13.18) [3]. Serum CN1 has a higher activity for carnosine compared to anserine which inhibits carnosine degradation by substrate inhibition [4]. In patients with type 2 diabetes mellitus (DM), especially in females, we [5,6,7] and others [8,9,10] demonstrated an association of the susceptibility to develop diabetic nephropathy (DN), with a variant in the carnosinase 1 gene (CNDP1), associated with lower carnosinase 1 (CN1) activity [11].

Carnosine inhibits glycation [12], acts as an ACE inhibitor [13,14], reduces oxidative damage and improves enzymatic and non-enzymatic antioxidant activity [15,16,17,18], while its carbonyl scavenging function is debated [19,20,21,22,23]. In rodents, carnosine supplementation consistently improved diabetic complications, e.g., diabetic nephropathy, retinopathy, neuropathy and wound healing [1,24,25,26,27,28,29]. The L-histidine residue, with its imidazole ring, exerts most of the buffering activity, i.e., metal chelation and antioxidant properties of carnosine. The carbonyl quenching and, in particular, its reactivity toward α,β-unsaturated carbonyls, requires a concerted mechanism involving both the primary amine and the imidazole ring [30]. Whereas carnosine has extensively been studied, little is known about the function of the methylated imidazole ring of anserine. Recent studies reported both dipeptides to prevent methylglyoxal (MG)-induced advanced glycation end product (AGE) and N epsilon-(Carboxyethyl)lysine (CEL) formation. There was a higher quenching activity of carnosine compared to anserine [30], and a lower anti-crosslinking property of anserine, as compared to carnosine [31]. Anti-radical capacity for neuronal cells was shown to be higher for anserine than carnosine [32]. In human kidneys, anserine levels are higher compared to carnosine, suggesting the important role of anserine in the kidney [33]. We investigated in vitro protective actions of anserine on different cellular stress tolerance markers, and the impact of exogenous anserine supplementation in diabetic mice.

## 2. Results

### 2.1. Effect of Anserine in Glucose-Stressed Tubular Cells

**Figure 1:**
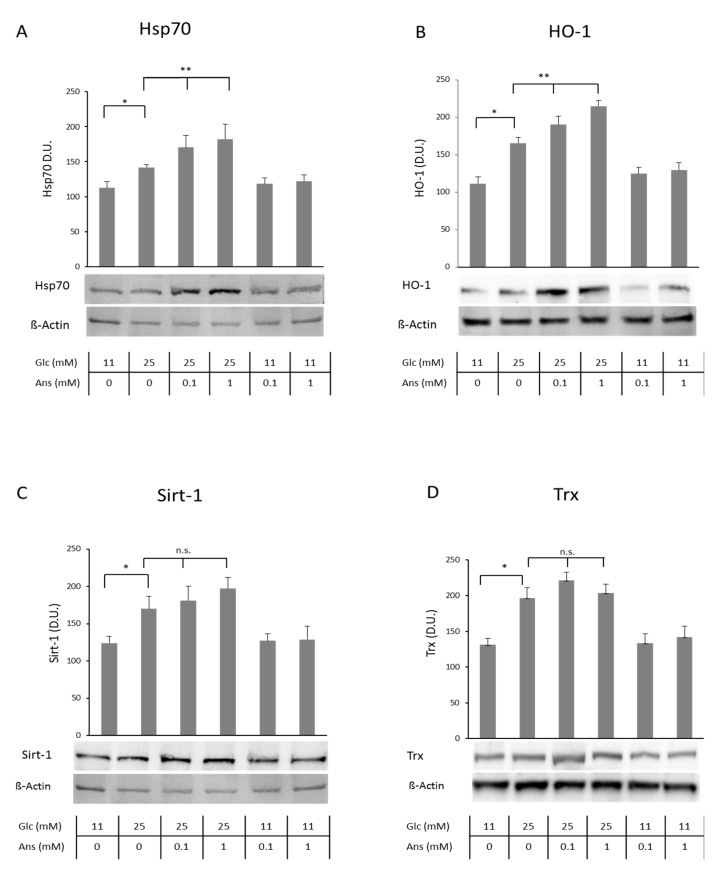
Effect of co-incubation with high glucose and anserine in human tubular cells (HK-2) on cellular heat shock protein 70 (Hsp70), hemeoxygenase (HO-1), Sirtuin-1 (Sirt-1) and Thioredoxin (Trx). Hsp70 (**A**), HO-1 (**B**), Sirt-1 (**C**) and Trx (**D**) cellular protein concentrations significantly increased in HK-2 cells with glucose stress (25 mM for 24 h), determined by Western blotting, compared to cells incubated with medium containing normal glucose concentration (11 mM). Densitometric units (D.U.) after normalization against β-actin are given (*n* = 3). Co-incubation with anserine (0.1 and 1 mM) further increased Hsp70 and HO-1 protein but had no additional effect on Sirt-1 and Trx. Anserine alone does not alter tubular cell defense systems. *p* < 0.05 (*); *p* < 0.01 (**); n.s. = not significantly.

**Figure 2:**
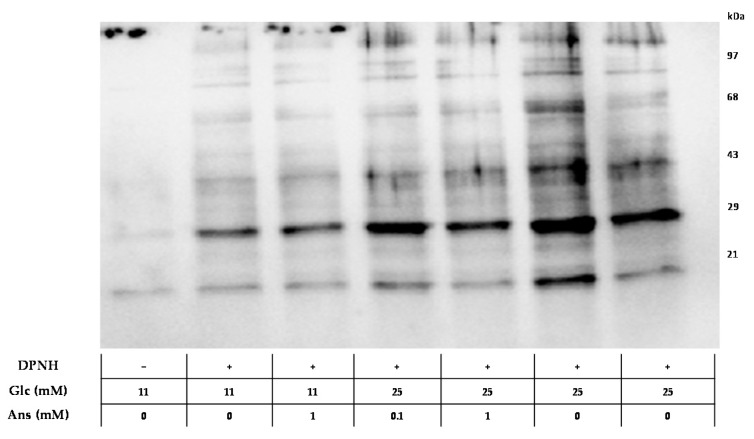
Effect of co-incubation with glucose and anserine in human tubular cells (HK-2) on total protein carbonylation. Glucose stress (25 mM) increased total protein carbonylation in HK-2 cells, compared to cells incubated under normal glucose concentration (11 mM; *n* = 3). Co-incubation with anserine (0.1 and 1 mM) reduced protein carbonylation. In unstressed cells, anserine had no effect on overall protein carbonylation. Protein carbonylation was visualized by derivatization with 2,4-dinitrophenolhydrazine (DNPH) and quantified immunochemically.

Glucose-stressed tubular cells (25 mM glucose for 24 h, compared to normal glucose concentration of 11 mM) increased cellular protein concentrations of sirtuin-1 (Sirt-1), thioredoxin (Trx), hemeoxygenase-1 (HO-1) and heat shock protein 70 (Hsp70), representing markers for cellular stress tolerance, compared to control and normalized to respective beta-actin concentrations (Figure 1A–D; all *p* < 0.05). Co-incubation with anserine (0.1 and 1 mM) further increased intracellular HO-1 and Hsp70 protein concentrations by about 30% (Figure 1A,B) but had no effect on Sirt-1 and Trx (Figure 1C,D). Increased protein carbonylation induced by glucose-stress could be dose-dependently reduced by co-incubation with 0.1 and 1 mM anserine (Figure 2).

### 2.2. Effect of Anserine in H_2_O_2_-Stressed Tubular Cells 

**Figure 3:**
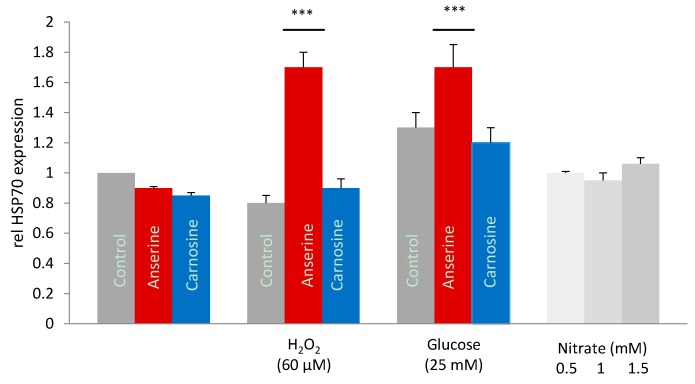
Effect of anserine and carnosine in human tubular cells exposed to oxidative and glycative stress. Human tubular cells (HK-2) were stressed by H_2_O_2_ (60 µM) and glucose (25 mM) and co-incubated with 1 mM anserine (red bars) and carnosine (blue bars), respectively, compared to control (grey bars). Cellular heat shock protein 70 (Hsp70) mRNA was measured by RT-PCR and normalized to expression of β-actin. Hsp70 expression significantly increased with co-incubation of anserine but not with carnosine. Since a nitrated form of anserine was applied, an independent effect of nitrate (green bars) on Hsp70 was ruled out. *p* < 0.01 (**); *p* < 0.001 (***).

Co-incubation of tubular cells with 40, 60 and 100 µM H_2_O_2_ and 1mM anserine, dose-dependently increased Hsp70 expression (normalized to β-actin). At 60 µM H_2_O_2_-exposure, anserine doubled Hsp70 mRNA in the tubular cells (1.7 ± 0.1 vs. 0.8 ± 0.05 relative to medium control; *p* < 0.001). In contrast, co-incubation of tubular cells with H_2_O_2_ and carnosine did not affect Hsp70 expression (0.9 ± 0.06; Figure 3). Since anserine was applied as nitrate salt, the addition of nitrate was tested. The addition of 0.5–1.5 mM nitrate had no effect on Hsp70 expression (Figure 3) and on HO-1 protein concentration (data not shown).

**Figure 4:**
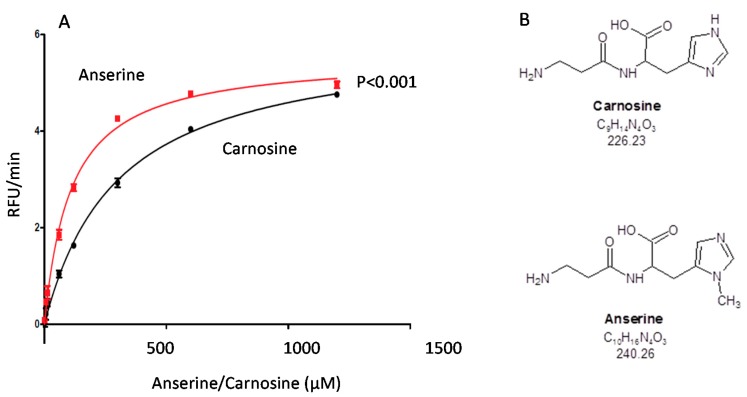
Antioxidant capacity of carnosine and anserine. (**A**) Antioxidant capacity of anserine and carnosine were assessed by a standardized oxygen radical absorbance capacity (ORAC) assay. Antioxidant capacity, given as relative fluorescence units (RFU), of anserine (red line) is significantly higher as compared to carnosine (black line). (**B**) Molecular structure of carnosine and its methylated derivate anserine.

Carnosine and anserine both exert antioxidant capacity in vitro, as determined by a standardized oxygen radical absorbance capacity assay. There is a higher capacity for anserine compared to carnosine at concentrations of 50–1000 µM for anserine and carnosine, which is within the range of (renal) tissue concentrations (*p* < 0.001, Figure 4)

### 2.3. Treatment of Diabetic Mice with Anserine

**Figure 5:**
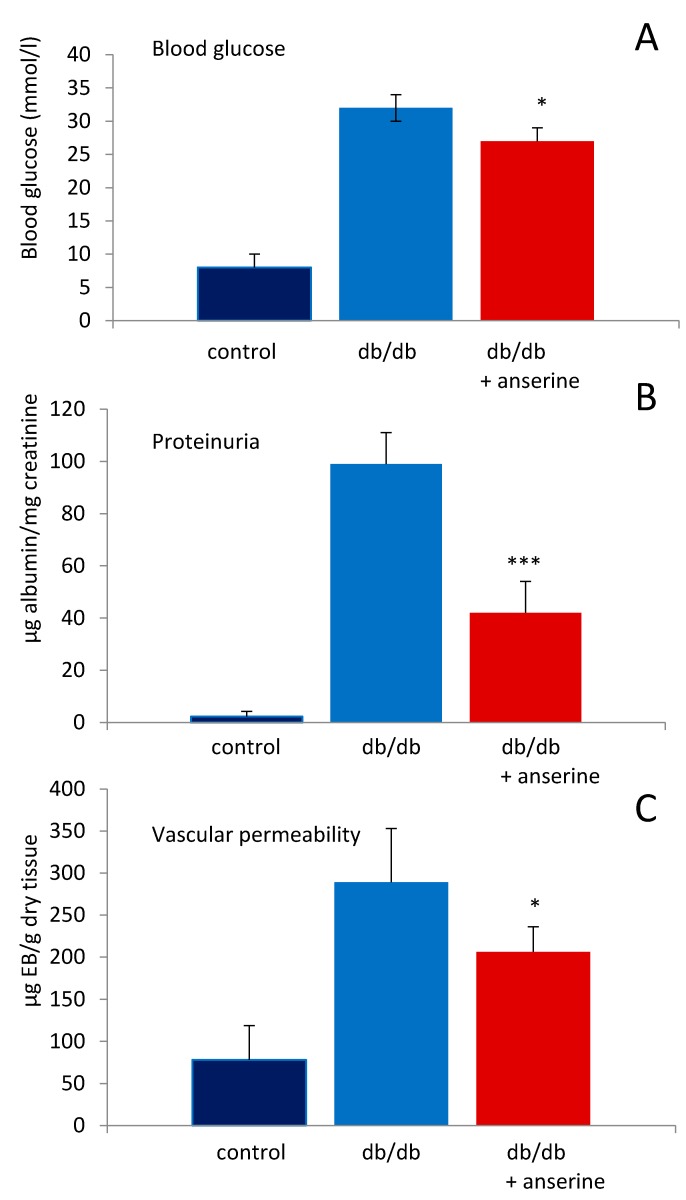
Effects of anserine on blood glucose, proteinuria and renal vascular permeability in diabetic mice (db/db). Twelve-week-old db/db were treated with three intravenous anserine injections every other day and scarified at week 14. Anserine treatment lowered blood glucose (**A**), proteinuria (**B**) and vascular permeability (**C**). *p* < 0.05 (*); *p* < 0.001 (***).

Twelve-week-old db/db and control mice (WT) were treated with three intravenous anserine injections every other day and sacrificed at week 14. At time of sacrifice, body weight was significantly higher in db/db versus WT mice (38 ± 5 g vs. 26 ± 1.8 g; *p* < 0.001). Anserine treatment had no effect on body weight in the db/db mice (39 ± 4 g) versus WT mice (27 ± 1.1 g; *p* = n.s). In the untreated db/db mice, blood glucose (32 ± 2 vs. 8 ± 0.6 mmol/L; *p* < 0.001), proteinuria (99 ± 36 vs. 2.3 ± 1.4 µg albumin/mg creatinine; *p* < 0.001) and vascular permeability (289 ± 64 vs. 78 ± 41 µg EB/g dry tissue; *p* < 0.001) were significantly increased compared to WT mice. Anserine treated db/db mice had significantly reduced blood glucose concentrations (32 ± 2 vs. 27 ± 2 mmol/L; *p* < 0.05), proteinuria (99 ± 36 vs. 42 ± 12 µg albumin/mg creatinine; *p* < 0.001) and less vascular Evans blue leakage, i.e., lower vascular permeability than untreated diabetic mice (289 ± 64 to 206 ± 30 µg EB/g dry tissue; *p* < 0.05; Figure 5). In non-diabetic WT mice, anserine had no effect on blood glucose (8 ± 0.6 vs. 8 ± 0.6 mmol/L; *p* = n.s.) or proteinuria (1.3 ± 0.6 vs. 2.3 ± 1.4 µg albumin/mg creatinine; *p* = n.s) compared to untreated WT mice.

## 3. Discussion

During recent years, several studies have demonstrated that carnosine supplementation in diabetic mice can improve glucose metabolism [27,34,35,36,37], proteinuria [23,25,27,35,38,39] and vascular permeability [39]. We now show that treatment of diabetic mice with only three doses of anserine (100 mg/kg every other day) improved blood glucose, proteinuria and vascular permeability. Anserine, but not carnosine, activates the intracellular Hsp70-defense system under oxidative and glycative stress.

Anserine and carnosine have both been reported to have quenching and antioxidant activity, but there are distinct differences in their protective properties. While both dipeptides can prevent methylglyoxal (MG)-induced AGE and CEL formation in vitro [40], the MG quenching activity is higher for carnosine compared to anserine [21,40]. The quenching activity of anserine and carnosine for malondialdehyde is in the same range, indicating that the imidazole ring is not involved in the quenching mechanism, as previously suggested [21]. Our comparison now demonstrates higher antioxidative activity for anserine compared to carnosine. Furthermore, our data suggests an important role of anserine, but not of carnosine, in activating the intracellular defense system under oxidative stress, by activating Hsp70 expression. Under glucose-induced stress, anserine also increased HO-1 concentration, a mediator of cyto- and tissue protection against a wide variety of injurious insults [41]. No effect on deacetylation by Sirt-1 or redoxsignalling via Trx was observed. Hsp70, HO-1, Sirt-1 and Trx are all part of the integrated system for cellular stress tolerance [28]. Furthermore, anserine could efficiently reduce protein carbonylation, the most severe modification induced in proteins by reactive oxygen species. Heat shock proteins, such as Hsp70, have multiple protective effects. They mediate a diverse range of cellular functions including: Folding and regulatory processes of cellular repair; interaction with cytoskeletal structures; participation in the transport of proteins through intracellular membranes of organelles; cleavage of protein aggregates [42,43] and the post-inflammation processes [44]. A lack of Hsp60 and Hsp70 induction in response to stress in target organs of diabetic complications has previously been reported. It has been postulated that the inability of the diabetic glomeruli to activate an effective stress response may contribute to particular susceptibility to diabetic injury [45].

Both anserine and carnosine supplementation in rodents have yielded an array of beneficial effects in diabetic mice. A direct comparison of either compound has not yet been performed but we have recently shown that carnosine supplementation in mice results in increased renal anserine concentrations [39]. We now demonstrate that only three doses of anserine in db/db mice substantially improves glucose homeostasis, reduces proteinuria by more than 50% and mitigates vascular leakage. Improved vascular leakage has been previously demonstrated for carnosine after long-term administration. Therefore, our findings suggest a highly potent protective action of anserine against renal long-term sequelae of diabetes. Further studies, however, directly comparing the effects of anserine and carnosine in vivo are mandatory, and studies elucidating whether the beneficial effects of carnosine treatment are, at least, partially caused by the protective effect of anserine. Noteworthy is that the nitrated form of anserine was administered in the mice. Although there is no evidence that nitrate improves proteinuria, future experiments should administer nitrate-free anserine, which has now become available. In our cell culture experiments, a role of nitrate on Hsp70 expression was excluded by the dose-dependent addition of nitrate. In humans, first intervention studies in pre-diabetic patients yielded promising results, although plasma half-life of carnosine is very short in humans. Since CN1 degradation rate is about 200-fold lower for anserine than for carnosine, anserine seems to be a promising therapeutic tool for humans [4].

In conclusion, we provide experimental evidence that anserine has a higher oxygen radical absorbance capacity than carnosine and that only anserine, but not carnosine, activates the intracellular defense system Hsp70. Our in vitro and in vivo findings point to a significant, and yet underexplored, renoprotective action of anserine in diabetes mellitus. The lower degradation rate of anserine compared to carnosine in human plasma underlines its potential role as a therapeutic target in humans.

## 4. Materials and Methods

### 4.1. Total Antioxidant Capacity

The standardized oxygen radical absorbance capacity (ORAC) assay was used to determine total antioxidant capacity. The thermal decomposition of 2,2-azobis(2-amidinopropane) dihydrochloride (AAPH) generates peroxyl radicals which quench the fluorescence signal of fluorescein. The addition of an antioxidant compound to the reaction stabilizes the fluorescence signal according to its antioxidant capacity.

### 4.2. Dipeptide Concentrations and CN1 Activity

Anserine and carnosine concentrations were measured fluorometrically by high-performance liquid chromatography, as previously described [46]. Briefly, deproteinized samples were derivatized with carbazole-9-carbonyl chloride (CFC), injected into the liquid chromatography and detected by fluorescence detection. The detection limit was 15 nM. All samples were measured twice and spiked with the standards to identify each analyte. CN1 activity was assayed according to a method described by Teufel et al. [4]. The reaction was initiated by the addition of carnosine or anserine to cell culture or tissue homogenate, and stopped by adding 1% trichloracetic acid. Liberated histidine was derivatized by adding o-Pthaldialdehyde (OPA) and fluorescence was read using a MicroTek plate reader (λExc: 360 nm; λEm: 460 nm).

### 4.3. Cell Culture

Immortalized human tubular cells (HK-2; American Type Culture Collection CRL-2190) were grown in RPMI 1640 medium with 10% fetal calf serum (*v*/*v*) and 1% penicillin and streptomycin (*v*/*v*) at 37 °C with 5% CO_2_.

### 4.4. Western Immunoblotting

The cells were homogenized in 0.1 M NaCl, 0.01 M Tris Cl (pH 7.6), 0.001 M EDTA (pH 8.0), 0.001 M PMSF, 1X Protease Inhibitor cocktail (Sigma, St. Louis, USA).) and the protein concentration was determined using the BCA method (Pierce Chemical, Dallas, TX, USA). Proteins extracted for each sample (50 µg protein per lane), were boiled for three minutes in sample buffer (2.5% SDS, 40 mM Tris-HCl, 5% 2-mercaptoethanol, 0.025 mg/mL of bromophenol blue, 5% glycerol) and then separated by SDS-PAGE in gels 4–20% (Bio-Rad Laboratories, Hercules, CA, USA) for 60 min at 100 V. Separated proteins were electrophoretically transferred to a nitrocellulose membrane (Bio-Rad Laboratories, Hercules, CA, USA) in a transfer buffer containing 192 mM glycine, 25 mM Tris, 0.05% SDS and 20% *v*/*v* methanol, for 1 h at 100 V. The transfer of the proteins to the nitrocellulose membrane was confirmed by staining with Ponceau, which was then removed by three washes in PBS (5 min each). Membranes were incubated for 1 h at room temperature (RT) in PBS and 0.1% Tween 20 (T-PBS) containing 2% milk powder. The membrane was probed with anti-Hsp70, anti-HO-1, anti-Trx and anti Sirt-1 polyclonal antibodies (Santa Cruz Biotech. Inc., CA, USA) overnight (4 °C in T-PBS) and incubated with a goat polyclonal antibody anti-beta-actin (SC-1615, Santa Cruz Biotech. Inc., CA, USA) for quantification. Excess unbound antibodies were removed. After incubation with the primary antibody, the membranes were washed (three times, 5 min each), incubated (for 1 h, RT) with the secondary polyclonal antibody coupled to a horseradish peroxidase enzyme. An appropriate luminescent substrate (SuperSignal detection system kit, Pierce Chemical, Dallas, TX, USA) was used, quantified (, Bioscience, London, UK), and analyzed with Molecular Imaging software. Each experiment was performed in triplicate and analyzed by one-way ANOVA, followed by the inspection of all differences by Duncan’s new multiple-range test and expressed as means ± S.E.M. Differences were considered significant at *p* < 0.05.

### 4.5. Western Blot of Carbonylated Proteins

Carbonylated proteins were analyzed using the OxyBlot kit according to the manufacturer’s instructions: OxyBlotTM Protein Oxidation (Merck Millipore, Darmstadt, Germany). Briefly, samples (15 µg protein) were denatured by adding 5 μL of 12% SDS to a final concentration of 6% SDS. The samples were derivatized by adding 10 μL of 1X DNPH (2,4-dinitrophenolhydrazine) solution and incubated (at RT for 15 min). Samples were neutralized (7.5 μL of neutralization solution) and the derivatized proteins were separated by SDS/PAGE (see above). The primary antibody used was against DNPH and detected by luminescence (SuperSignal detection system kit: Pierce Chemical, Dallas, TX, USA). The bands were quantified (Gel-Logic 2200-PRO Bioscience, London, UK), and analyzed (Molecular Imaging software).

### 4.6. Expression of Heat Shock Protein 70

qPCR was performed using DyNAmo ColorFlash SYBR Green qPCR Master Mix (Thermo Fisher Scientific, Waltham, MA, USA) and a LightCycler^®^ 480 Instrument II (Roche Diagnostics, Indianapolis, IN, USA). Signals of amplified products were verified using melting curve analysis and mRNA levels were normalized to β-actin. Relative expression levels were calculated using the Ct method described elsewhere [45]. Primer sequences used for analyzing mRNA content were: Hsp70 (PrimerBankID: 387211a2), forward 5′- GAGATCGACTCTCTGTTCGAGG-3′ and reverse 5′- GCCCGTTGAAGAAGTCCTG -3′ β-Actin (PrimerBank ID: 6671509a1), forward 5′- GGCTGTATTCCCCTCCATCG -3′ and reverse 5′- CCAGTTGGTAACAATGCCATGT -3’.

### 4.7. Db/db Mice

Male C57BL/KsJm/Leptdb (db/db) mice (Stock 000662) and their normoglycemic heterozygous littermates were obtained from Charles River (Sulzfeld, Germany, Stock 000662). The animals were housed in a 12-h light/dark cycle at 22 °C and provided ad libitum with standard laboratory food and water. The experimental procedure was approved by the North Stockholm Ethical Committee for Care and Use of Laboratory Animals. Glucose levels were determined at the end of the experiment in blood collected from the tail tip (by OneTouch Ultra Blood Glucose meter; LifeScan, Milpitas, CA, USA). Material from 5 animals was used for each measurement.

### 4.8. Anserine Treatment

Treatment with anserine started at 12 weeks. The animals were divided into 4 groups, each consisting of 5 animals: (1) control mice with no treatment, (2) control mice who received 3 anserine dose intravenous injections of 100 mg/kg every other day, (3) db/db mice with no treatment and (4) db/db mice that received (Sigma, Stockholm, Sweden) 3 anserine intravenous injections of 100 mg/kg every other day (i.e., every 48 h). At week 14, the mice were sacrificed.

### 4.9. Animal Rights 

The experimental procedure was approved by the North Stockholm Ethical Committee for Care and Use of Laboratory Animals (N78/10, date of approval: 18.03.2010).

### 4.10. Proteinuria

The animals were euthanized by carbon dioxide after the treatment period. Proteinuria was measured in spot urine collected at the end of the treatment period. Urinary albumin was determined by indirect competitive ELISA assay (Exocell, Lallaing, France). Creatinine was quantitated by a chemical analysis based on Jaffe´s reaction of alkaline picrate with creatinine (Exocell, Lallaing, France).

### 4.11. Tissue and Blood Sampling

The animals were euthanized by carbon dioxide. The kidneys were removed, immediately homogenized in an ice-cold buffer containing 20 mM HEPES, 210 mM mannitol and 70 mM sucrose per gram tissue, pH 7.2. The homogenate was centrifuged at 1500× *g* for 5 min at 4 °C, and the supernatant was kept at −80 °C until analysis.

### 4.12. Vascular Permeability Assay

Vascular permeability was assessed by measurement of Evans blue leakage from the kidney vessels into the neighboring tissue. A 1% solution of Evans blue dye (2 µL/mg BW, Sigma Chemical, St. Louis, MI, USA) was injected into the tail vein of db/db mice, treated or not with anserine. After 10 min the mice were euthanized by CO_2_ inhalation, blood samples were collected and the kidneys were removed, blotted dry, and weighed. The Evans blue dye was extracted from the kidney with 1 mL of formamide overnight at 65 °C and measured spectrophotometrically at 620 nm [47,48].

### 4.13. Statistical Analysis

Data were obtained from at least 3 independent experiments and quantitative data are given as mean and standard deviation (SD). Student t-test was calculated to compare groups. A *p*-value of <0.05 was considered significant. Significance in experiments comparing more than two groups was evaluated by one-way analysis of variance, followed by post hoc analysis using Tukey’s test.

## References

[1] Boldyrev A.A., Aldini G., Derave W. (2013). Physiology and pathophysiology of carnosine. Physiol. Rev..

[2] Kwiatkowski S., Kiersztan A., Drozak J. (2018). Biosynthesis of carnosine and related dipeptides in vertebrates. Curr. Protein Pep. Sci..

[3] Teufel M., Saudek V., Ledig J.P., Bernhardt A., Boularand S., Carreau A., Cairns N.J., Carter C., Cowley D.J., Duverger D. (2003). Sequence identification and characterization of human carnosinase and a closely related non-specific dipeptidase. J. Biol. Chem..

[4] Peters V., Jansen E.E., Jakobs C., Riedl E., Janssen B., Yard B.A., Wedel J., Hoffmann G.F., Zschocke J., Gotthardt D. (2011). Anserine inhibits carnosine degradation but in human serum carnosinase (CN1) is not correlated with histidine dipeptide concentration. Clin. Chim. Acta.

[5] Janssen B., Hohenadel D., Brinkkoetter P., Peters V., Rind N., Fischer C., Rychlik I., Cerna M., Romzova M., de Heer E. (2005). Carnosine as a protective factor in diabetic nephropathy: Association with a leucine repeat of the carnosinase gene CNDP1. Diabetes.

[6] Albrecht T., Zhang S., Braun J.D., Xia L., Rodriquez A., Qiu J., Peters V., Schmitt C.P., van den Born J., Bakker S.J.L. (2017). The CNDP1 (CTG)5 Polymorphism Is Associated with Biopsy-Proven Diabetic Nephropathy, Time on Hemodialysis, and Diabetes Duration. J. Diabetes Res..

[7] Mooyaart A., van Valkengoed I.G., Shaw P.K., Peters V., Baelde H.J., Rabelink T.J., Bruijn J.A., Stronks K., de Heer E. (2009). Lower frequency of the 5/5 homozygous CNDP1 genotype in South Asian Surinamese. Diabetes Res. Clin. Pract..

[8] Ahluwalia T.S., Lindholm E., Groop L.C. (2011). Common variants in CNDP1 and CNDP2, and risk of nephropathy in type 2 diabetes. Diabetologia.

[9] Alkhalaf A., Landman G.W., van Hateren K.J., Groenier K.H., Mooyaart A.L., De Heer E., Gans R.O., Navis G.J., Bakker S.J., Kleefstra N. (2015). Sex specific association between carnosinase gene CNDP1 and cardiovascular mortality in patients with type 2 diabetes (ZODIAC-22). J. Nephrol..

[10] Freedman B.I., Hicks P.J., Sale M.M., Pierson E.D., Langefeld C.D., Rich S.S., Xu J., McDonough C., Janssen B., Yard B.A. (2007). A leucine repeat in the carnosinase gene CNDP1 is associated with diabetic end-stage renal disease in European Americans. Nephrol. Dial. Transpl..

[11] Peters V., Zschocke J., Schmitt C.P. (2018). Carnosinase, diabetes mellitus and the potential relevance of carnosinase deficiency. J. Inherit. Metab. Dis..

[12] Alhamdani M., Al-Azzawie H.F., Abbas F.K. (2007). Decreased formation of advanced glycation end-products in peritoneal fluid by carnosine and related peptides. Perit. Dial. Int..

[13] Hou W., Chen H.J., Lin Y.H. (2003). Antioxidant peptides with Angiotensin converting enzyme inhibitory activities and applications for Angiotensin converting enzyme purification. J. Agric. Food Chem..

[14] Nakagawa K., Ueno A., Nishikawa Y. (2006). Interactions between carnosine and captopril on free radical scavenging activity and angiotensin-converting enzyme activity in vitro. Yakugaku Zasshi.

[15] Decker E.A., Livisay S.A., Zhou S. (2000). A re-evaluation of the antioxidant activity of purified carnosine. Biochemistry.

[16] Velez S., Nair N.G., Reddy V.P. (2008). Transition metal ion binding studies of carnosine and histidine: Biologically relevant antioxidants. Colloids Surf B Biointerfaces.

[17] Hipkiss A.R. (2011). Energy metabolism, proteotoxic stress and age-related dysfunction—Protection by carnosine. Mol. Aspects Med..

[18] Babizhayev M.A., Lankin V.Z., Savel’Yeva E.L., Deyev A.I., Yegorov Y.E. (2013). Diabetes mellitus: Novel insights, analysis and interpretation of pathophysiology and complications management with imidazole-containing peptidomimetic antioxidants. Recent Pat. Drug Deliv. Formul..

[19] Barski O.A., Xie Z., Baba S.P., Sithu S.D., Agarwal A., Cai J., Bhatnagar A., Srivastava S. (2013). Dietary carnosine prevents early atherosclerotic lesion formation in apolipoprotein E.-null mice. Arterioscler. Thromb. Vasc. Biol..

[20] Brings S., Fleming T., De Buhr S., Beijer B., Lindner T., Wischnjow A., Kender Z., Peters V., Kopf S., Haberkorn U. (2017). A scavenger peptide prevents methylglyoxal induced pain in mice. Biochim. Biophys. Acta.

[21] Vistoli G., Colzani M., Mazzolari A., Gilardoni E., Rivaletto C., Carini M., Aldini G. (2017). Quenching activity of carnosine derivatives towards reactive carbonyl species: Focus on alpha-(methylglyoxal) and beta-(malondialdehyde) dicarbonyls. Biochem. Biophys. Res. Commun..

[22] Colzani M., De Maddis D., Casali G., Carini M., Vistoli G., Aldini G. (2016). Reactivity, Selectivity, and Reaction Mechanisms of Aminoguanidine, Hydralazine, Pyridoxamine, and Carnosine as Sequestering Agents of Reactive Carbonyl Species: A. Comparative Study. Chem. Med. Chem..

[23] Aldini G., Orioli M., Rossoni G., Savi F., Braidotti P., Vistoli G., Yeum K.J., Negrisoli G., Carini M. (2011). The carbonyl scavenger carnosine ameliorates dyslipidaemia and renal function in Zucker obese rats. J. Cell Mol. Med..

[24] Ansurudeen I., Sunkari V.G., Grunler J., Peters V., Schmitt C.P., Catrina S.B., Brismar K., Forsberg E.A. (2012). Carnosine enhances diabetic wound healing in the db/db mouse model of type 2 diabetes. Amino Acids.

[25] Iacobini C., Menini S., Blasetti Fantauzzi C., Pesce C.M., Giaccari A., Salomone E., Lapolla A., Orioli M., Aldini G., Pugliese G. (2018). FL-926-16, a novel bioavailable carnosinase-resistant carnosine derivative, prevents onset and stops progression of diabetic nephropathy in db/db mice. Br. J. Pharmacol..

[26] Riedl E., Pfister F., Braunagel M., Brinkkotter P., Sternik P., Deinzer M., Bakker S.J., Henning R.H., van den Born J., Kramer B.K. (2011). Carnosine prevents apoptosis of glomerular cells and podocyte loss in STZ diabetic rats. Cell Physiol. Biochem.

[27] Peters V., Riedl E., Braunagel M., Hoger S., Hauske S., Pfister F., Zschocke J., Lanthaler B., Benck U., Hammes H.P. (2014). Carnosine treatment in combination with ACE inhibition in diabetic rats. Regul. Pept..

[28] Bellia F., Calabrese V., Guarino F., Cavallaro M., Cornelius C., De Pinto V., Rizzarelli E. (2009). Carnosinase levels in aging brain: Redox state induction and cellular stress response. Antioxid. Redox. Signal..

[29] Pfister F., Riedl E., Wang Q., vom Hagen F., Deinzer M., Harmsen M.C., Molema G., Yard B., Feng Y., Hammes H.P. (2011). Oral carnosine supplementation prevents vascular damage in experimental diabetic retinopathy. Cell Physiol. Biochem..

[30] Vistoli G., Colzani M., Mazzolari A., Maddis D.D., Grazioso G., Pedretti A., Carini M., Aldini G. (2016). Computational approaches in the rational design of improved carbonyl quenchers: Focus on histidine containing dipeptides. Future Med. Chem..

[31] Hobart L.J., Seibel I., Yeargans G.S., Seidler N.W. (2004). Anti-crosslinking properties of carnosine: Significance of histidine. Life Sci..

[32] Boldyrev A., Bulygina E., Leinsoo T., Petrushanko I., Tsubone S., Abe H. (2004). Protection of neuronal cells against reactive oxygen species by carnosine and related compounds. Comp. Biochem. Physiol. B Biochem. Mol. Biol..

[33] Peters V., Klessens C.Q., Baelde H.J., Singler B., Veraar K.A., Zutinic A., Drozak J., Zschocke J., Schmitt C.P., de Heer E. (2015). Intrinsic carnosine metabolism in the human kidney. Amino Acids.

[34] Sauerhofer S., Yuan G., Braun G.S., Deinzer M., Neumaier M., Gretz N., Floege J., Kriz W., van der Woude F., Moeller M.J. (2007). L-carnosine, a substrate of carnosinase-1, influences glucose metabolism. Diabetes.

[35] Albrecht T., Schilperoort M., Zhang S., Braun J.D., Qiu J., Rodriguez A., Pastene D.O., Kramer B.K., Koppel H., Baelde H. (2017). Carnosine Attenuates the Development of both Type 2 Diabetes and Diabetic Nephropathy in BTBR ob/ob Mice. Sci. Rep..

[36] Nagai K., Tanida M., Niijima A., Tsuruoka N., Kiso Y., Horii Y., Shen J., Okumura N. (2012). Role of L-carnosine in the control of blood glucose, blood pressure, thermogenesis, and lipolysis by autonomic nerves in rats: Involvement of the circadian clock and histamine. Amino Acids.

[37] Lee Y.T., Hsu C.C., Lin M.H., Liu K.S., Yin M.C. (2005). Histidine and carnosine delay diabetic deterioration in mice and protect human low density lipoprotein against oxidation and glycation. Eur. J. Pharmacol..

[38] Menini S., Iacobini C., Ricci C., Scipioni A., Blasetti Fantauzzi C., Giaccari A., Salomone E., Canevotti R., Lapolla A., Orioli M. (2012). D-Carnosine octylester attenuates atherosclerosis and renal disease in ApoE null mice fed a Western diet through reduction of carbonyl stress and inflammation. Br. J. Pharmacol..

[39] Peters V., Schmitt C.P., Zschocke J., Gross M.L., Brismar K., Forsberg E. (2012). Carnosine treatment largely prevents alterations of renal carnosine metabolism in diabetic mice. Amino Acids.

[40] Weigand T., Singler B., Fleming T., Nawroth P., Klika K.D., Thiel C., Baelde H., Garbade S.F., Wagner A.H., Hecker M. (2018). Carnosine Catalyzes the Formation of the Oligo/Polymeric Products of Methylglyoxal. Cell Physiol. Biochem..

[41] Ryter S.W., Alam J., Choi A.M. (2006). Heme oxygenase-1/carbon monoxide: From basic science to therapeutic applications. Physiol. Rev..

[42] Beck F.X., Neuhofer W., Muller E. (2000). Molecular chaperones in the kidney: Distribution, putative roles, and regulation. Am. J. Physiol. Renal. Physiol..

[43] Chebotareva N., Bobkova I., Shilov E. (2017). Heat shock proteins and kidney disease: Perspectives of HSP therapy. Cell Stress Chaperones.

[44] Bellia F., Vecchio G., Cuzzocrea S., Calabrese V., Rizzarelli E. (2011). Neuroprotective features of carnosine in oxidative driven diseases. Mol. Aspects Med..

[45] Barutta F., Pinach S., Giunti S., Vittone F., Forbes J.M., Chiarle R., Arnstein M., Perin P.C., Camussi G., Cooper M.E. (2008). Heat shock protein expression in diabetic nephropathy. Am. J. Physiol. Renal Physiol..

[46] Peters V., Lanthaler B., Amberger A., Fleming T., Forsberg E., Hecker M., Wagner A.H., Yue W.W., Hoffmann G.F., Nawroth P. (2015). Carnosine metabolism in diabetes is altered by reactive metabolites. Amino Acids.

[47] Ibla J.C., Khoury J. (2006). Methods to assess tissue permeability. Methods Mol. Biol..

[48] Matthew C.B., Sils I.V., Bastille A.M. (2002). Tissue-specific extravasation of albumin-bound Evans blue in hypothermic and rewarmed rats. Can. J. Physiol. Pharmacol..

